# Inorganic phosphate self-sufficient whole-cell biocatalysts containing two co-expressed phosphorylases facilitate cellobiose production

**DOI:** 10.1093/jimb/kuac008

**Published:** 2022-03-15

**Authors:** Lei Wang, Peng Zheng, Meirong Hu, Yong Tao

**Affiliations:** Chinese Academy of Sciences Key Laboratory of Microbial Physiological and Metabolic Engineering, Institute of Microbiology, Chinese Academy of Sciences, Beijing 100101, China; College of Life Science, University of Chinese Academy of Sciences, Beijing 100049, China; Chinese Academy of Sciences Key Laboratory of Microbial Physiological and Metabolic Engineering, Institute of Microbiology, Chinese Academy of Sciences, Beijing 100101, China; State Key Laboratory of Food Science and Technology, Nanchang University, Nanchang 330047, China; Chinese Academy of Sciences Key Laboratory of Microbial Physiological and Metabolic Engineering, Institute of Microbiology, Chinese Academy of Sciences, Beijing 100101, China; Chinese Academy of Sciences Key Laboratory of Microbial Physiological and Metabolic Engineering, Institute of Microbiology, Chinese Academy of Sciences, Beijing 100101, China; College of Life Science, University of Chinese Academy of Sciences, Beijing 100049, China

**Keywords:** Cellobiose, Phosphorylase, Co-expression, Inorganic phosphate, Whole-cell biocatalyst

## Abstract

Cellobiose, a natural disaccharide, attracts extensive attention as a potential functional food/feed additive. In this study, we present an inorganic phosphate (Pi) self-sufficient biotransformation system to produce cellobiose by co-expressing sucrose phosphorylase (SP) and cellobiose phosphorylase (CBP). The *Bifidobacterium adolescentis* SP (BASP) and *Cellvibrio gilvus* CBP (CGCBP) were co-expressed in *Escherichia coli. Escherichia coli* cells containing BASP and CGCBP were used as whole-cell catalysts to convert sucrose and glucose to cellobiose. The effects of reaction pH, temperature, Pi concentration, and substrate concentration were investigated. In the optimum biotransformation conditions, 800 mM cellobiose was produced from 1.0 M sucrose, 1.0 M glucose, and 50 mM Pi, within 12 hr. The by-product fructose and residual substrate (sucrose and glucose) were efficiently removed by treatment with yeast, to help purify the product cellobiose. The wider applicability of this Pi self-sufficiency strategy was demonstrated in the production of laminaribiose by co-expressing SP and laminaribiose phosphorylase. This study suggests that the Pi self-sufficiency strategy through co-expressing two phosphorylases has the advantage of great flexibility for enhanced production of cellobiose (or laminaribiose).

## Introduction

Oligosaccharides are important for a wide range of applications in the food, medicine, nutrition, and cosmetic industries. One interesting example of disaccharides with physiological functions or potential prebiotic effects is cellobiose (van Zanten et al., 2015). Cellobiose, a zero-calorie functional sweetener, attracts considerable interest because of its biological functions (Schwaiger et al., 2020). As a dietary fiber, cellobiose is a biologically functional feed additive that is well tolerated by animals (Nakamura et al., 2004). For example, the observed increase of *Clostridium* in the feces of horses may indicate beneficial and potentially prebiotic effects of cellobiose (Paßlack et al., 2020). The effect of cellobiose supplementation on the performance, health status, and digestive traits of growing rabbits has been evaluated (Ocasio-Vega et al., 2019; Zhong et al., 2019). Moreover, *Bifidobacterium infantis* remains viable during 4 weeks of storage in milk supplemented with cellobiose, suggesting the usefulness of cellobiose as a prebiotic ingredient in fermented products involving bifidobacteria (Basholli-Salihu et al., 2013). When cellobiose is ingested by humans, there is no increase in blood glucose or insulin secretion, and orally ingested cellobiose is well fermented in the large intestine (Ruiz-Matute et al., 2011). Consequently, cellobiose is an emerging food and feed ingredient, large-scale production of which by biocatalytic methods has received considerable attention in recent years (Brucher & Häßler, 2019; Ubiparip et al., 2020).

Cellobiose is predominately obtained on an industrial scale by acid hydrolysis of cellulose, the abundant organic compound in nature (Zhong & Nidetzky, 2020). However, it is difficult to isolate cellobiose effectively because of the by-products cello-oligosaccharides and glucose (Homma et al., 1993). Cellulases cleave the second 1,4-linkages from reducing or nonreducing ends of cellulose to release cellobiose, but the activities of these enzymes are not sufficient for the industrial production of cellobiose (Ubiparip et al., 2020). Cellobiose has been enzymatically synthesized from starch using glucan phosphorylase and cellobiose phosphorylase (CBP; EC 2.4.1.20), but the process involves three steps and has a yield of just 23.7% (Suzuki et al., 2009). Furthermore, the production of cellobiose from sucrose using three enzymes, sucrose phosphorylase (SP; EC 2.4.1.7), xylose isomerase, and CBP, has been reported, but only 62.3 mM cellobiose was obtained from 100 mM sucrose (Zhong et al., 2017). Previous studies have indicated that the key precursor for cellobiose production is α-glucose-1-phosphate (G-1-P). G-1-P can be produced from two primary carbohydrates, sucrose and starch, by phosphorylases (Winter et al., 2011; Zhong et al., 2017). SP is a key enzyme that catalyzes the phosphorolysis of sucrose. In the presence of inorganic phosphate (Pi), SP can convert sucrose to produce G-1-P and the by-product fructose (Goedl et al., 2007; Kitaoka et al., 1992a). CBP is classified as disaccharide phosphorylase, which catalyzes reversible phosphorolysis of the corresponding disaccharides into G-1-P and glucose (Kitaoka et al., 2012; Yoshida et al., 1998). CBP belongs to the CAZy (a database of carbohydrate-active enzymes) family GH94, together with chitobiose phosphorylase and cellodextrin phosphorylase. The natural role of CBP is the energy-efficient metabolism of cellobiose, producing G-1-P in which much of the energy of the substrate is conserved (Cagnin et al., 2019; de Groeve et al., 2011; Yuan & Zhao, 2013).

**Fig. 1.**
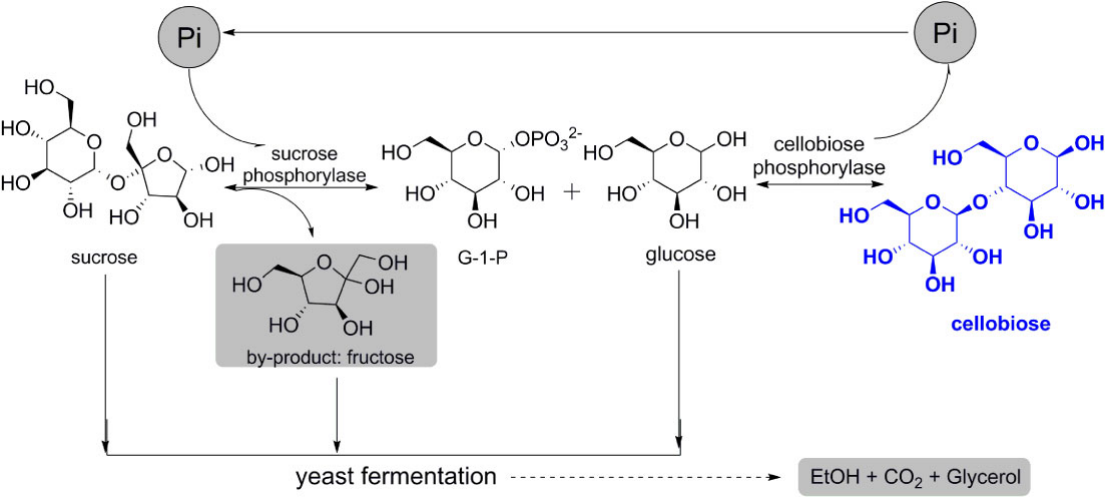
Inorganic phosphate (Pi) self-sufficient biotransformation system to produce cellobiose from sucrose and glucose by co-expressing sucrose phosphorylase and cellobiose phosphorylase. The by-product (fructose) and residual substrate (sucrose and glucose) were removed by yeast fermentation.

In the presence of Pi, the cascade reaction of SP and CBP converts sucrose and glucose into cellobiose through a two-step glucosyl transfer via G-1-P (Brucher & Häßler, 2019; Zhong et al., 2017). Therefore, the primary factors influencing cellobiose biosynthesis are the high activity of SP and CBP, and the concentration of Pi. However, a high Pi concentration has an important influence on cellobiose synthesis due to product inhibition and causes phosphorus pollution of the environment (Ubiparip et al., 2020; Zhong et al., 2017, 2020). As such, a Pi self-sufficient biotransformation method to produce cellobiose from sucrose and glucose by co-expressing SP and CBP was developed. The by-product fructose and residual substrate were removed by yeast fermentation (Fig. 1). We also developed an effective synthesis of laminaribiose (β-1,3-linked glucobiose) from sucrose and glucose using this Pi self-sufficient strategy, by co-expressing SP and laminaribiose phosphorylase (LBP; EC 2.4.1.31). The strategy of Pi self-sufficient biotransformation by co-expressing two phosphorylases is a promising method to meet industrial requirements for cellobiose (or laminaribiose) production.

## Materials and Methods

### Bacterial Strains, Plasmids, and Chemicals

All strains and plasmids used in this work are listed in Table 1. *Escherichia coli* Trans T1 was used for the construction and propagation of the plasmid, while *E. coli* BW25113 was the host for enzyme expression. The vector pYB1s (derived from the pBAD/HisB vector) was used to provide the pBAD promoter and the corresponding rrnB terminator. All genes encoding SP, CBP, and LBP were artificially synthesized by General Biosystems Co., Ltd (Anhui, China). Sucrose, glucose, and fructose were purchased from Shanghai ShengGong Bio-chemical Co. Ltd (Shanghai, China). Standards for G-1-P, cellobiose, laminaribiose, and other chemicals were purchased from Aladdin (Shanghai, China).

**Table 1.** Bacterial strains and plasmids used in this study

Strain or plasmid	Description	Source
Strain		
Trans T1	Wild type	This laboratory
BW25113	Wild type	This laboratory
BASP	BW25113 harboring pYB1s-BASP	This study
BLSP	BW25113 harboring pYB1s-BLSP	This study
LCSP	BW25113 harboring pYB1s-LCSP	This study
CGCBP	BW25113 harboring pYB1s-CGCBP	This study
CTCBP	BW25113 harboring pYB1s-CTCBP	This study
EGLBP	BW25113 harboring pYB1s-EGLBP	This study
PSLBP	BW25113 harboring pYB1s-PSLBP	This study
HTLBP	BW25113 harboring pYB1s-HTLBP	This study
CB01	BW25113 harboring pYB1s-BASP-CGCBP	This study
CB02	BW25113 harboring pYB1s-CGCBP-BASP	This study
LR01	BW25113 harboring pYB1s-PSLBP-BASP	This study
Plasmids		
pYB1s	Expression vector	Laboratory
pYB1s-BASP	pYB1s containing BASP gene from *Bifidobacterium adolescentis*	This study
pYB1s-BLSP	pYB1s containing TCADC gene from *Bifidobacterium longum*	This study
pYB1s-LCSP	pYB1s containing ECAspA gene from *Leuconostoc citreum*	This study
pYB1s-CGCBP	pYB1s containing CGCBP gene from *Cellvibrio gilvus*	This study
pYB1s-CTCBP	pYB1s containing CTCBP gene from *Clostridium thermocellum*	This study
pYB1s-EGLBP	pYB1s containing EGLBP gene from *Euglena gracilis*	This study
pYB1s-PSLBP	pYB1s containing PSLBP gene from *Paenibacillus* sp.	This study
pYB1s-HTLBP	pYB1s containing HTLBP gene from *Halorhabdus tiamatea*	This study
pYB1s-BASP-CGCBP	pYB1s containing BASP gene from *B. adolescentis*, and CGCBP gene from *C. gilvus*	This study
pYB1s-CGCBP-BASP	pYB1s containing CGCBP gene from *C. gilvus*, and BASP gene from *B. adolescentis*	This study
pYB1s-PSLBP-BASP	pYB1s containing PSLBP gene from *Paenibacillus* sp., and BASP gene from *B. adolescentis*	This study

### Media and Cultivation Conditions

Luria–Bertani (LB) medium (10 g/l tryptone, 5 g/l yeast extract, and 10 g/l sodium chloride) was used for plasmid construction. During plasmid construction, cultures were grown at 37°C on a shaker at 220 rpm in LB medium with streptomycin (50 μg/ml) added as required. For enzyme expression, overnight cultures were inoculated into ZYM autoinduction medium (1% tryptone, 0.5% yeast extract, 25 mM Na_2_HPO_4_, 25 mM KH_2_PO_4_, 50 mM NH_4_Cl, 5 mM Na_2_SO_4_, 2 mM MgSO_4_, 0.5% glycerol, 0.05% glucose, 0.2% lactose, and trace metals). ZYM trace metals (1000 ×) contained 50 mM FeCl_3_, 20 mM CaCl_2_, 10 mM each of MnCl_2_ and ZnSO_4_, and 2 mM each of CoCl_2_, CuCl_2_, NiCl_2_, Na_2_MoO_4_, Na_2_SeO_3_, and H_3_BO_3_ (Studier, 2005).

### Heterologous Expression of Phosphorylases

Engineered *E. coli* strains containing different phosphorylases were cultured in 5 ml of LB medium with appropriate antibiotics at 37°C for 6–7 hr. One milliliter of seed culture was then transferred to a 500-ml flask containing 100 ml of ZYM autoinduction medium. Cultures were incubated in a rotary shaker at 220 rpm and 37°C until the absorbance at 600 nm (OD_600_ _nm_) was between 0.6 and 0.8, at which point phosphorylase expression was induced at 30°C for 12 hr. The phosphorylases used in this study were SP from *Bifidobacterium adolescentis* (BASP), CBP from *Cellvibrio gilvus* (CGCBP), and LBP from *Paenibacillus* sp. YM1 (PSLBP). The expression of phosphorylases was confirmed by sodium dodecyl sulfate–polyacrylamide gel electrophoresis (SDS-PAGE).

### Construction of Whole-Cell Catalysts for Cellobiose and Laminaribiose Production

The main primers used in this study are listed in Table S1. The PCR products were purified, linked with vector pYB1s, and transformed into *E. coli* Trans-T1 for sequencing. For the construction of pYB1s-BASP-CGCBP, pYB1s-CGCBP-BASP, and pYB1s-PSLBP-BASP, PCR products (CGCBP- and BASP-encoding gene fragments) were inserted into pYB1s-BASP, pYB1s-CGCBP, and pYB1s-PSLBP, respectively, by using the Gibson assembly method (Gibson et al., 2009). The resulting expression vectors pYB1s-BASP-CGCBP, pYB1s-CGCBP-BASP, and pYB1s-PSLBP-BASP were transformed into *E. coli* BW25113 to construct whole-cell biocatalysts, named CB01, CB02, and LR01, respectively.

### High-level Production of Cellobiose and Laminaribiose by Co-Expressing Two Phosphorylases

Under the optimized reaction conditions, cellobiose was produced from sucrose and glucose by using the recombinant strain CB01, which co-expressed BASP and CGCBP. Cells of strain CB01 were harvested by centrifugation, adjusted to OD_600_ _nm_ = 30, and then resuspended in 50 mM phosphate buffer (pH 6.5) containing 2 mM MgCl_2_, 1 M sucrose, and 1 M glucose. The bioconversion was performed in a 1-l bioreactor with 500 ml working volume.

For laminaribiose production, cells of strain LR01, which co-expressed BASP and PSLBP, were harvested by centrifugation at 6,000 × *g* for 10 min. The conversion experiments were carried out in a 1-l bioreactor with 500 ml working volume. The whole-cell biocatalyst LR01 (OD_600_ _nm_ = 30) and 2 mM MgCl_2_ were added to the reaction system, the buffer was 50 mM phosphate buffer (pH 6.5), and the substrates were 1 M sucrose and 1 M glucose.

### Analytical Methods

Cell density was estimated by measuring the optical density at 600 nm. The expression of recombinant phosphorylases was analyzed by SDS-PAGE. The concentrations of cellobiose, glucose, G-1-P, Pi, fructose, and glycerol were measured by HPLC (Agilent 1260 series, Hewlett Packard) with an Aminex HPX-87H column (7.8 × 300 mm). The analysis was performed at 50°C with a mobile phase comprising 5 mM H_2_SO_4_ at a flow rate of 0.3 ml/min by a refractive index detector (RID). The HPLC chromatograms of cellobiose, glucose, G-1-P, Pi, fructose, and glycerol are shown in Fig. S1.

## Results and Discussion

### Expression and Selection of Phosphorylases

**Fig. 2.**
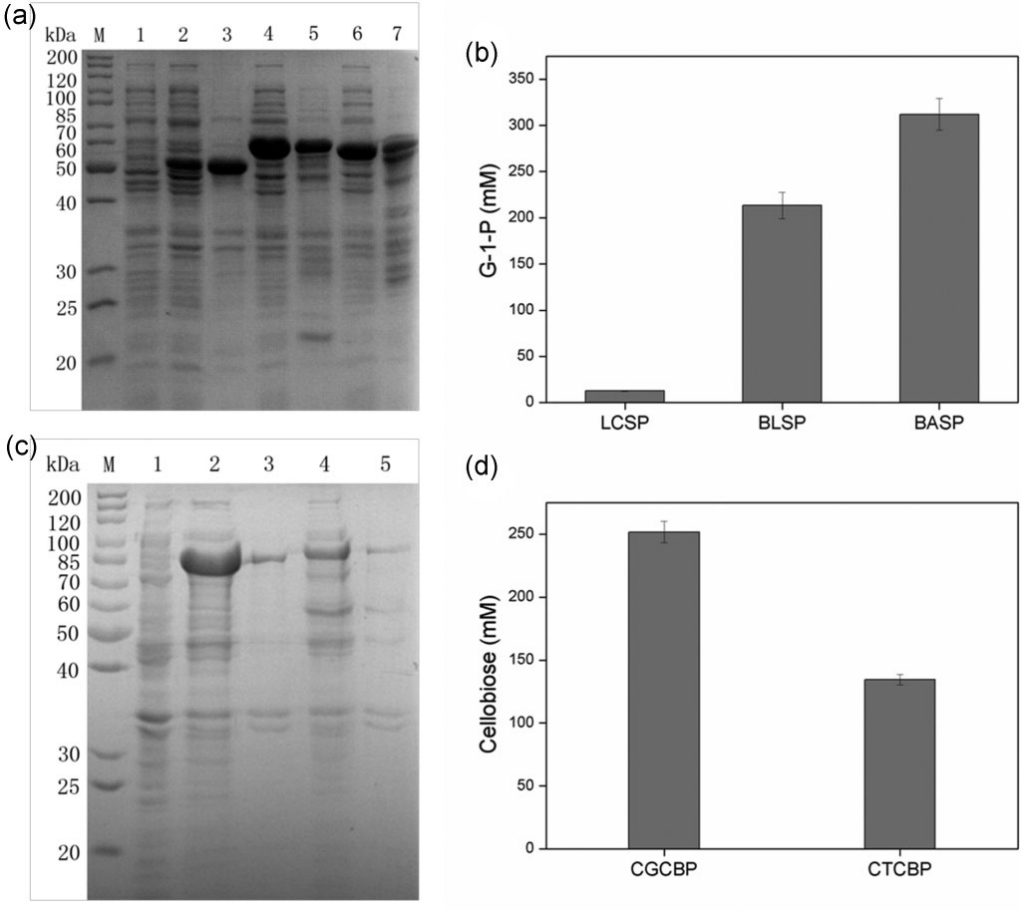
Expression and selection of SP and CBP enzymes. The predicted molecular weights of SP and CBP enzymes are shown in Table S2. (a) SDS-PAGE analysis of the SP enzymes. Lane M, protein marker; lane 1, control; lane 2, soluble extract of *E. coli* expressing LCSP; lane 3, insoluble extract of *E. coli* expressing LCSP; lane 4, soluble extract of *E. coli* expressing BASP; lane 5, insoluble extract of *E. coli* expressing BASP; lane 6, soluble extract of *E. coli* expressing BLSP; lane 7, insoluble extract of *E. coli* expressing BLSP. (b) G-1-P production from sucrose and Pi by SP enzymes from different species. Reaction conditions: biomass OD_600 nm_ = 20, sucrose 0.5 M, sodium phosphate buffer 0.5 M (pH 6.0, Pi 0.5 M), 40°C, reaction time 12 hr. (c) SDS-PAGE analysis of the CBP enzymes. Lane M, protein marker; lane 1, control; lane 2, soluble extract of *E. coli* expressing CGCBP; lane 3, insoluble extract of *E. coli* expressing CGCBP; lane 4, soluble extract of *E. coli* expressing CTCBP; lane 5, insoluble extract of *E. coli* expressing CTCBP. (d) Cellobiose production from G-1-P and glucose by CBP enzymes from different species. Reaction conditions: biomass OD_600 nm_ = 20, G-1-P 0.5 M, glucose 0.5 M, Pi 0 mM, sodium acetate/acetate buffer (pH 5.0), 40°C, reaction time 12 hr. Error bars indicate standard deviations of three independent assays.

SP is a key transglucosidase that catalyzes the reversible conversion of sucrose (a primary product of plant photosynthesis) and phosphate into G-1-P and d-fructose (Franceus & Desmet, 2020; Goedl et al., 2010; Gudiminchi & Nidetzky, 2017). The production of G-1-P from sucrose by three SPs from different species was investigated. BASP (GenBank: AF543301.1) from *B. adolescentis*, BLSP (GenBank: KP136872.1) from *B. longum*, and LCSP (GenBank: NZ_CP042410.1) from *Leuconostoc citreum* were respectively overexpressed in *E. coli* (Fig. 2a). BASP effectively catalyzed the conversion of sucrose and phosphate into G-1-P; ∼330 mM G-1-P was obtained from 0.5 M sucrose and 0.5 M Pi (Fig. 2b). Thus, BASP was selected for the production of G-1-P.

Cellobiose can be produced by CBP-catalyzed synthetic reaction of glucose and G-1-P to release Pi (Groeve et al., 2011). The CBPs from *Clostridium thermocellum, Ruminococcus flavefaciens*, and *C. gilvus* have been applied for cellobiose production (Kitaoka et al., 1993; Zhang & Lynd, 2006). CTCBP (GenBank: AY072794.1) from *C. thermocellum* and CGCBP (GenBank: BAA28631.1) from *C. gilvus* were respectively overexpressed in *E. coli* (Fig. 2c). CGCBP exhibited high catalytic activity in the production of cellobiose, and ∼250 mM cellobiose was obtained from 0.5 M G-1-P and 0.5 M glucose (Fig. 2d). Therefore, CGCBP was chosen for cellobiose production.

### Construction of Whole-Cell Biocatalysts Co-Expressing BASP and CGCBP

**Fig. 3.**
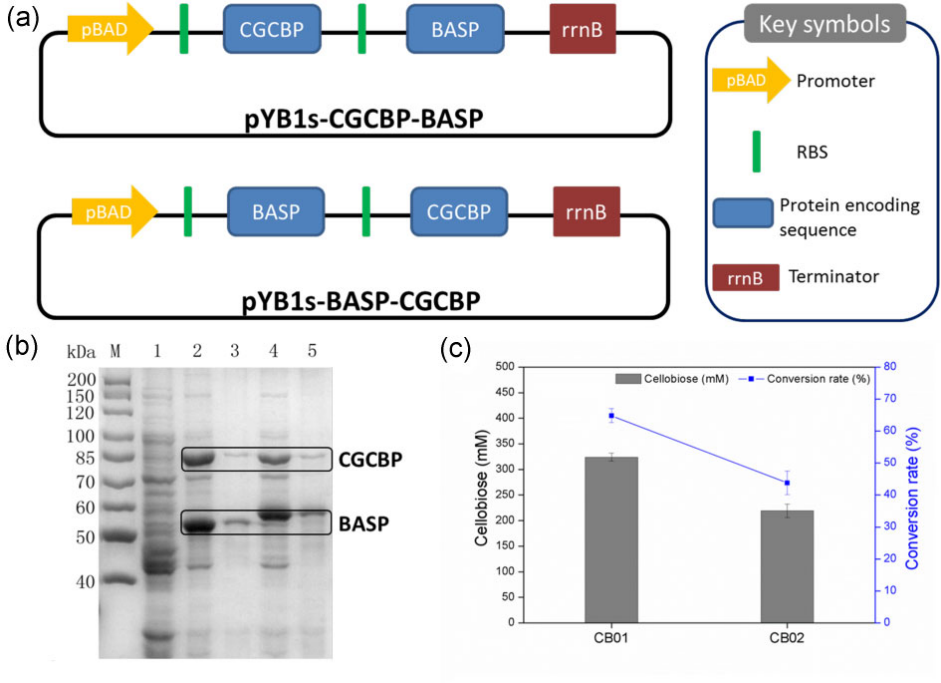
Selection of whole-cell biocatalysts containing co-expressed BASP and CGCBP. (a) The dual plasmid configurations for co-expression of BASP and CGCBP. (b) SDS-PAGE analysis of co-expressed BASP and CGCBP. Lane M, protein marker; lane 1, control; lane 2, soluble extract of CB01 (*E. coli*/pYB1s-CGCBP-BASP); lane 3, insoluble extract of CB01; lane 4, soluble extract of CB02 (*E. coli*/pYB1s-BASP-CGCBP); lane 5, insoluble extract of CB02. (c) Cellobiose production from sucrose and glucose by using whole-cell biocatalysts CB01 and CB02. Reaction conditions: biomass OD_600 nm_ = 20, sucrose 0.5 M, glucose 0.5 M, sodium phosphate buffer 50 mM (pH 6.0, Pi 50 mM), 40°C, reaction time 12 hr. Error bars indicate standard deviations of three independent assays.

Whole-cell biotransformation using recombinant strains containing a cascade of enzymes is an attractive method to produce many chemicals, and the biocatalytic reactions using whole-cell biocatalysts have the advantages of low cost, better process control, environmental friendliness, and greater efficiency (de Carvalho, 2017; Morgado et al., 2016; Xu et al., 2021). As such, BASP and CGCBP were co-expressed under the control of the promoter pBAD in *E. coli* using the plasmid pYB1s. Plasmids pYB1s-BASP-CGCBP and pYB1s-CGCBP-BASP were constructed to co-express the two phosphorylases to determine the effects of the order of the genes (Fig. 3a). The plasmids pYB1s-BASP-CGCBP and pYB1s-CGCBP-BASP were respectively transformed into *E. coli* to obtain whole-cell biocatalysts named CB01 and CB02. The expression of BASP and CGCBP was analyzed by SDS-PAGE (Fig. 3b). To determine the catalytic efficiency of the two whole-cell biocatalysts, cells of CB01 and CB02 were grown, harvested by centrifugation, and adjusted to OD_600_ _nm_ = 20 in 50 mM phosphate buffer (pH 6.0). The cellobiose synthesis reaction was performed at 40°C, and the substrate concentrations were 0.5 M sucrose and 0.5 M glucose. CB01 exhibited high performance in producing cellobiose from sucrose and glucose; ∼330 mM cellobiose was obtained from 0.5 M sucrose and 0.5 M glucose with a conversion rate of 65% (Fig. 3c).

BASP (from *B. adolescentis*) and CGCBP (from *C. gilvus*) were selected for co-expression in *E. coli* BW25113 for the production of cellobiose. The BASP protein showed a higher molecular weight in SDS-PAGE (Fig. 3b, lanes 4–5) when the genes encoding BASP and CGCBP were placed in pYB1s in the order BASP-CGCBP (lanes 4–5) than when they were in the order CGCBP-BASP (lanes 2–3). In the former arrangement, the *N*-terminus of BASP encoded in pYB1s included a sequence that translates into the extra amino acids MGTSSSGLVPRGSLE. Although BASP and CGCBP co-expressed in one plasmid in either order exhibited high expression levels (Fig. 3b), strain CB01 with plasmid pYB1s-CGCBP-BASP was more effective in the production of cellobiose (Fig. 3c). The low production of cellobiose by using strain CB02 might be due to the incomplete folding of enzymes during transcription and translation, suggesting that the order of the two genes might affect the apparent activities of the two enzymes. Hence, the order of the genes should be considered when multiple enzymes are co-expressed from one plasmid. In addition, the CGCBP protein showed a higher expression level in SDS-PAGE (Fig. 3b, lanes 2–5) when two genes were placed in pYB1s in the order CGCBP-BASP (strain CB01) than when they were in the order BASP-CGCBP (strain CB02). These results showed that CB01 (gene order CGCBP-BASP) exhibited high performance in producing cellobiose (Fig. 3c). Therefore, CGCBP is a key enzyme for the Pi self-sufficient biotransformation method to produce cellobiose from sucrose and glucose.

### Optimization of Reaction Parameters for Cellobiose Production

In this study, the one-pot biotransformation of sucrose and glucose to produce cellobiose involves two reversible reactions: (1) phosphorylation of sucrose to G-1-P and fructose catalyzed by BASP and (2) synthesis of cellobiose from G-1-P and glucose catalyzed by CGCBP. Pi plays an important role because it is a substrate of SP for G-1-P generation and a by-product of cellobiose synthesis by CBP. A high Pi concentration promotes the generation of G-1-P by SP, but Pi also decreases the reaction rate of cellobiose synthesis because of product inhibition (Zhong et al., 2017, 2020). Moreover, suitable catalytic conditions enable whole-cell biocatalysts to perform effectively, and pH and temperature are important factors that greatly affect such bioconversions (Xu et al., 2021). As such, the effects of the substrate molar ratio, Pi concentration, pH, and temperature were investigated to obtain the maximum production of cellobiose.

**Fig. 4.**
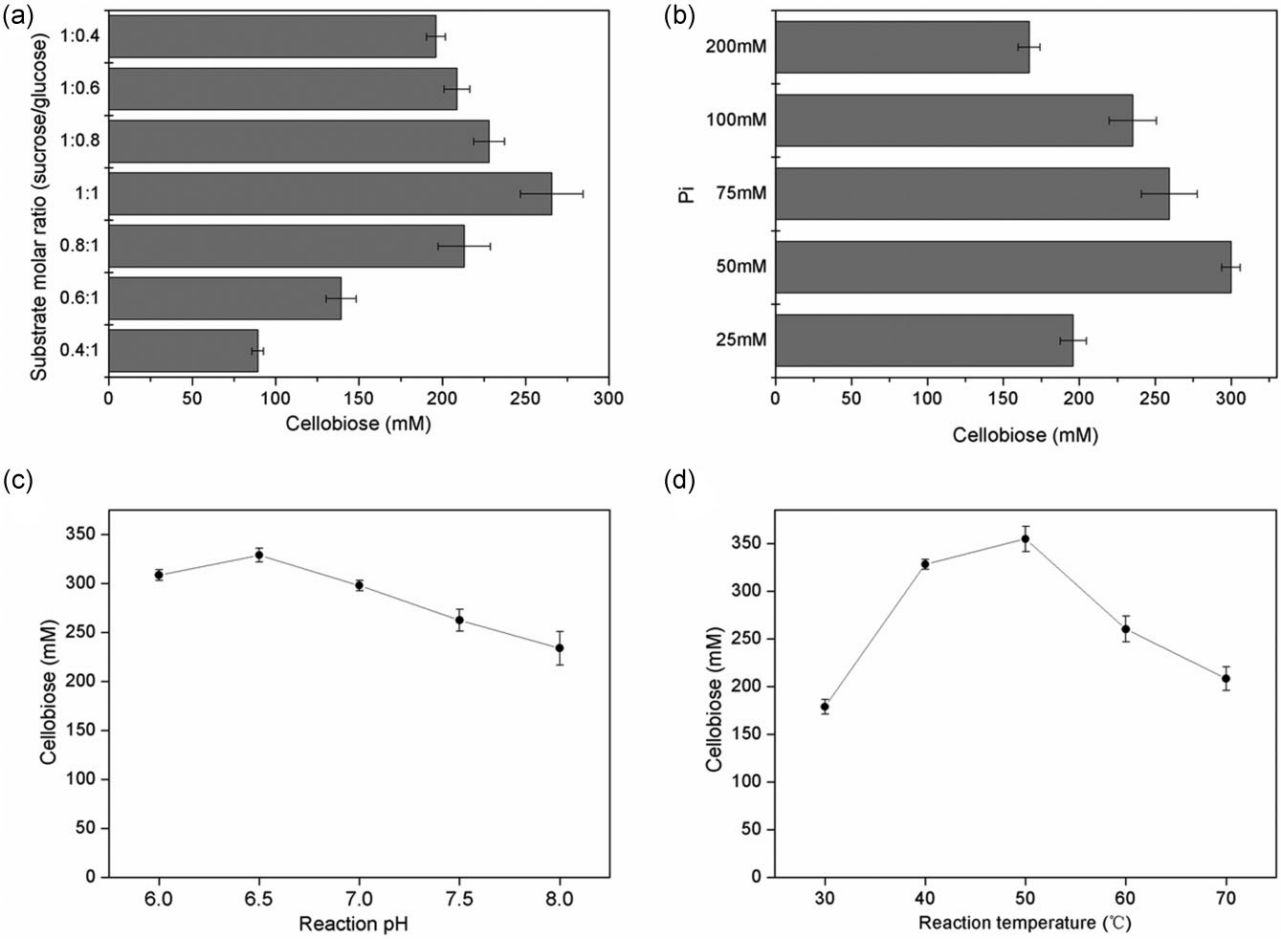
Optimization of reaction parameters for cellobiose production. (a) Effect of substrate molar ratio on cellobiose production. (b) Effect of Pi concentration on cellobiose production. (c) Optimization of pH for cellobiose production. (d) Optimization of temperature for cellobiose production. Reaction conditions: biomass OD_600 nm_ = 20, sucrose 0.2–0.5 M, glucose 0.2–0.5 M, sodium phosphate buffer (pH 6.0–8.0, Pi 25–200 mM), 30–70°C, reaction time 12 hr. Error bars indicate standard deviations of three independent assays.

In this system developed in this work, the supply of Pi was maintained at a nearly constant level, and glucose is needed to start the cellobiose production reaction, and the kinetic requirements of CBP mean that glucose is added at the beginning of the two-step reaction (Suzuki et al., 2009). We firstly carried out the effects of substrate molar ratio and Pi concentration for cellobiose production. The substrate molar ratio (sucrose:glucose) was set to 1:0.4, 1:0.6, 1:0.8, 1:1, 0.8:1, 0.6:1, and 0.4:1. The maximum amount of cellobiose was obtained with a molar ratio of 1:1 ([sucrose] and [glucose] = 0.5 M) (Fig. 4a). This condition was used for subsequent screening of the best Pi concentration, and the reaction pH and temperature. A Pi concentration of 50 mM (in sodium phosphate buffer) resulted in the maximum yield of cellobiose (Fig. 4b). Sodium phosphate buffers (pH 6.0–8.0) were used to test the effects of the reaction pH; as shown in Fig. 4c, the optimum pH for cellobiose production was 6.5. The reaction was performed at 30–70°C to investigate the effect of temperature. The maximum cellobiose concentration (350 mM) was obtained from 0.5 M sucrose and 0.5 M glucose at 50°C (Fig. 4d).

### Enhanced Cellobiose Production by Adjusting Substrate Concentrations and Biomass of CB01

Prior studies largely focused on enzymatic production of cellobiose by using multienzyme transformations or immobilized bienzymatic reactions (Brucher & Häßler, 2019; Schwaiger et al., 2020). However, these titers and the substrate conversion rates are not approaching the levels required for industrialization. Therefore, it is necessary to enhance cellobiose production to meet industrial requirements.

**Fig. 5.**
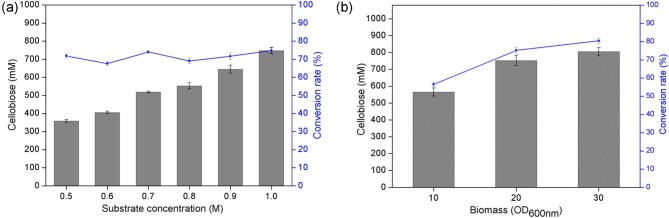
Enhanced cellobiose production by adjusting substrate concentrations and biomass of CB01. (a) Effect of substrate concentration on the production of cellobiose. (b) Effect of biomass of CB01 on the production of cellobiose. Reaction conditions: biomass OD_600 nm_ = 10, 20, 30, sucrose 0.5–1.0 M, glucose 0.5–1.0 M, sodium phosphate buffer 50 mM (pH 6.5, Pi 50 mM), 50°C, reaction time 12 hr. Error bars indicate standard deviations of three independent assays.

In this study, we develop a Pi self-sufficient whole-cell catalytic system for cellobiose production from sucrose and glucose. The substrate (sucrose and glucose) concentrations were increased from 0.5 to 1.0 M to enhance cellobiose production by the whole-cell biocatalyst CB01. The concentration of cellobiose obtained reached 750 mM (conversion rate 75%) (Fig. 5a). Under this condition, the molar ratios of these reactions were all 1:1, in which the concentrations of sucrose and glucose were from 0.5 to 1.0 M, and the appropriate reaction time was 12 hr (Fig. S2). The optimum biomass of CB01 for cellobiose production was investigated. Keeping the sucrose and glucose concentrations at 1.0 M, the biomass of CB01 was set at OD_600_ _nm_ = 10, 20, or 30. Finally, the conversion rate reached 80% with CB01 biomass of OD_600_ _nm_ = 30, and the concentration of cellobiose obtained was 800 mM (Fig. 5b).

### Removal of By-product and Residual Substrate by Treatment with Yeast

In the whole-cell biotransformation process, the by-product fructose (∼180 g/l) originated from sucrose, and residual glucose (∼40 g/L) was retained in the reaction system. The by-product fructose and residual substrate (glucose and sucrose) could be efficiently removed by treatment with yeast (Winter et al., 2011). Therefore, the reaction solutions were treated with yeast to remove the by-product fructose and residual substrate to help purify the cellobiose. We screened the ability of different yeast strains to consume fructose. Strain SC01 effectively consumed 100 g/L fructose in 12 hr (Fig. S4A). Next, consumption of cellobiose, sucrose, and glucose by strain SC01 was tested; sucrose (100 g/L) or glucose (100 g/l) was consumed completely within 24 hr, but cellobiose (100 g/l) was not degraded by SC01 (Fig. S4B). As such, the reaction solution from whole-cell biotransformations of glucose and sucrose to produce cellobiose was treated with SC01 to aid with the purification of cellobiose.

After treatment with SC01, the by-product fructose and residual substrate glucose were degraded, there was no residual sucrose, and trace glycerol was observed (Fig. S5). The yeast cells were removed by centrifugation, and the supernatant was treated using cation exchange resin JK800 and anion exchange resin D392 (Xi'an Lanxiao Technology New Material Co., Ltd) to remove ions. Then, the solution was decolored by adding activated charcoal (30 g/l) in an 80:20 water/ethanol solution. The trace glycerol adsorbed onto the activated charcoal. The water/ethanol solutions were pooled and concentrated under a vacuum at 50°C to obtain mother liquor, which was crystallized to obtain cellobiose crystals. As shown in Table S3, the final purity of cellobiose crystals reached 95%.

### Production of Laminaribiose from Sucrose and Glucose by Pi Self-Sufficient System Containing Two Phosphorylases

Laminaribiose has attracted widespread attention as a soluble dietary fiber, a powerful germination agent, and a widely used antiseptic (Kitaoka et al., 1992b; Müller et al., 2017a; Sun et al., 2019). Laminaribiose is also valuable because of its promising roles in biotechnology, including as a precursor for building blocks in the pharmaceutical industry, and as an inducer substrate for developing a regulatable gene expression system in *C. thermocellum* (Mearls et al., 2015). Conventional laminaribiose production involves chemical hydrolysis of laminarin, but this has disadvantages, including low product yields, high consumption of energy and water, and high separation costs (Kitaoka et al., 1992b). A previous study reported that 31.9 g/l laminaribiose was obtained from sucrose and glucose in bienzymatic batch experiments with reaction-integrated product separation by adsorption, but the low yield and the cumbersome procedure limit the application of this approach (Müller et al., 2017b).

**Fig. 6.**
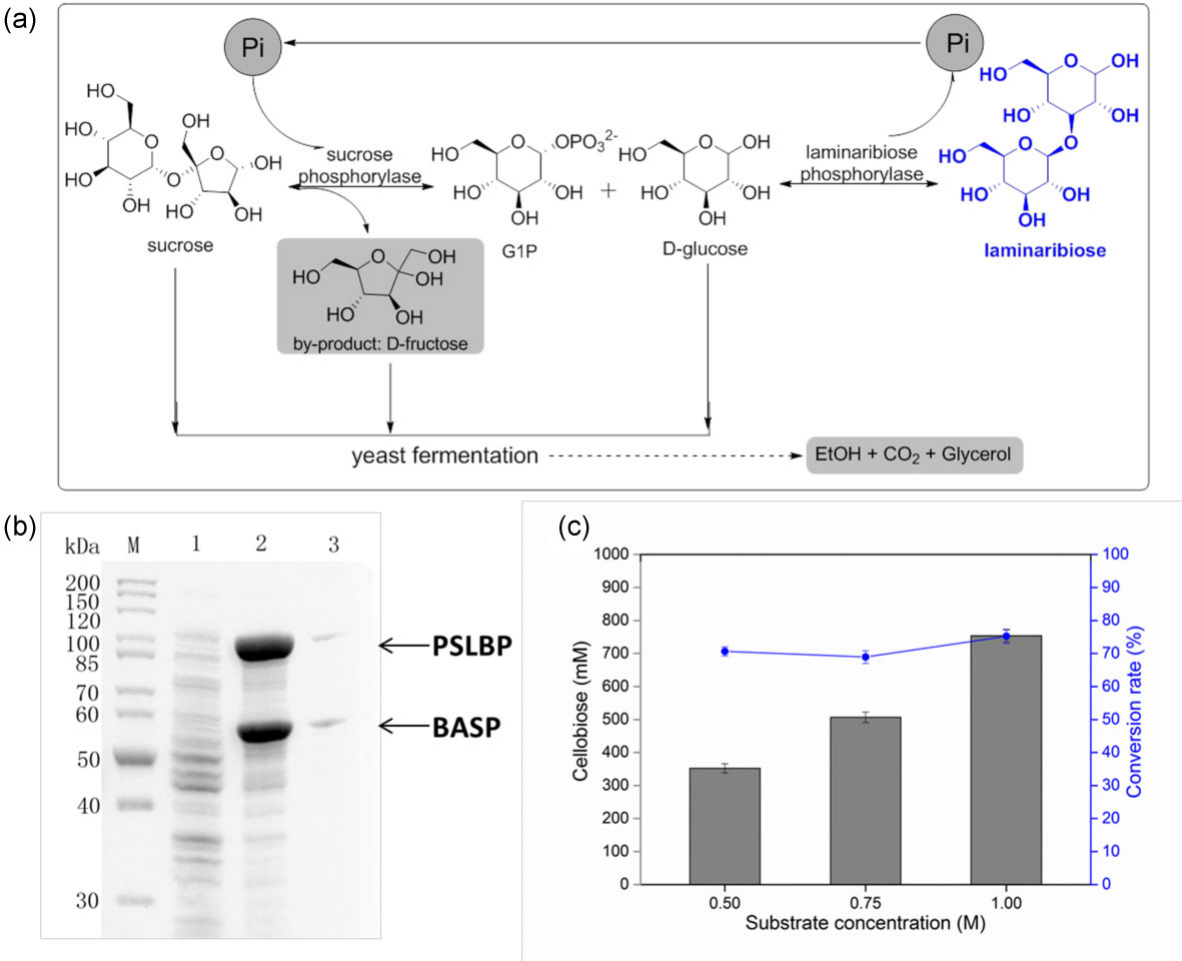
Production of laminaribiose from sucrose and glucose by a Pi self-sufficient whole-cell biocatalyst containing two phosphorylases. (a) Pi self-sufficient biotransformation system to produce laminaribiose from sucrose and glucose by co-expressing sucrose phosphorylase and laminaribiose phosphorylase. (b) SDS-PAGE analysis of whole-cell biocatalyst LR01 co-expressing BASP and PSLBP. Lane M, protein marker; lane 1, control; lane 2, soluble extract of LR01 (*E. coli*/pYB1s-PSLBP-BASP); lane 3, insoluble extract of LR01. (c) Effect of substrate concentration on the production of laminaribiose. Reaction conditions: OD_600 nm_ = 30, sucrose 0.5, 0.75, and 1.0 M, glucose 0.5, 0.75, and 1.0 M, sodium phosphate buffer 50 mM (pH 6.5, Pi 50 mM), 50°C, reaction time 12 hr. Error bars indicate standard deviations of three independent assays.

LBP catalyzes the production of laminaribiose from glucose and G-1-P (Kitaoka et al. 2012). Here, PSLBP (GenBank: BAJ10826.1) from *Paenibacillus* sp. YM1, EGLBP (GenBank: AUO30192.1) from *Euglena gracilis*, and HTLBP (GenBank: CCQ33042.1) from *Halorhabdus tiamatea* were, respectively, overexpressed in *E. coli* (Fig. S3A). Approximately 250 mM laminaribiose was obtained from 0.5 M G-1-P and 0.5 M glucose using PSLBP (Fig. S3B). As such, PSLBP and BASP were co-expressed from a pYB1s-based plasmid, which was transformed into *E. coli* to generate the whole-cell biocatalyst LR01, to produce laminaribiose from sucrose and glucose (Fig. 6a). The expression of BASP and PSLBP was analyzed by SDS-PAGE (Fig. 6b). Then, laminaribiose was produced using the whole-cell biocatalyst LR01, which co-expressed BASP and PSLBP by this Pi self-sufficient strategy. The biotransformation conditions for laminaribiose production were as follows: molar ratio of sucrose and glucose 1:1, reaction pH 6.5 (sodium phosphate buffer, 50 mM), and reaction temperature 50°C. The maximum laminaribiose concentration obtained (750 mM) was achieved with substrate concentrations of 1.0 M sucrose and 1.0 M glucose (conversion rate 75%) (Fig. 6c).

## Conclusion

**Fig. 7.**
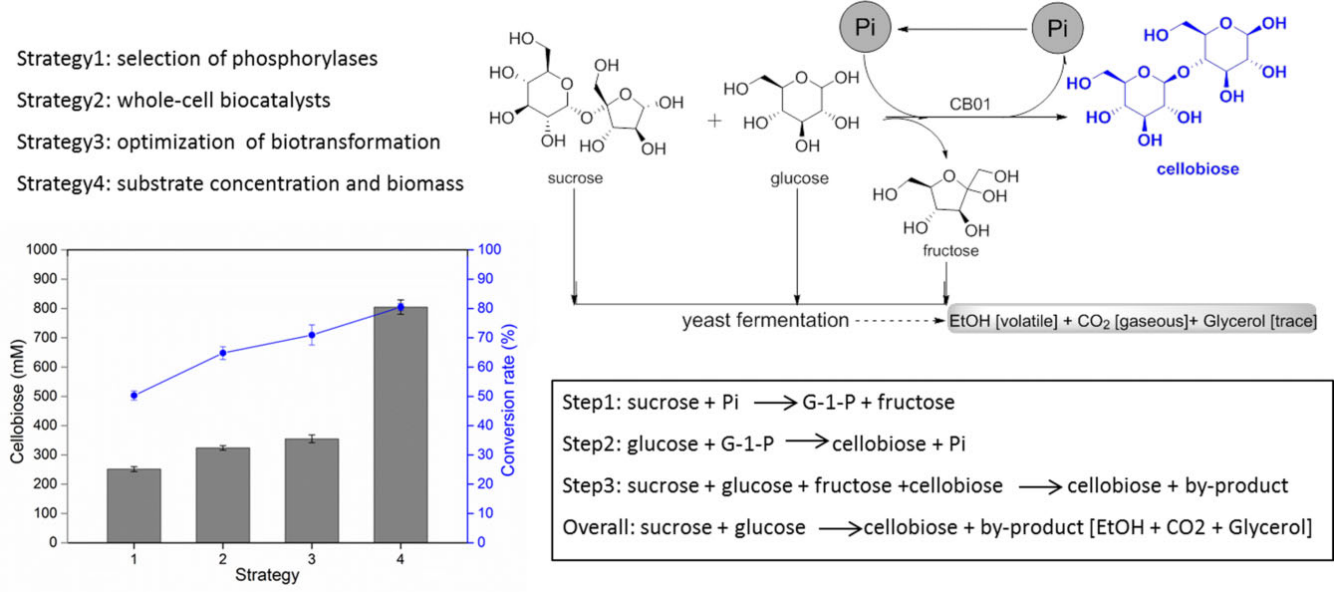
Summary of whole-cell biotransformation procedures for cellobiose production from sucrose and glucose by a Pi self-sufficient whole-cell biocatalyst CB01 containing two phosphorylases.

In conclusion, a Pi self-sufficient whole-cell catalytic system was developed for cellobiose production by co-expressing the phosphorylases SP and CBP in *E. coli*. The resulting whole-cell biocatalyst CB01 was used to produce cellobiose from sucrose and glucose; after optimization, the titer of cellobiose reached 275 g/l (an 80% substrate conversion rate) (Fig. 7). To demonstrate the wider applicability of this strategy, we also produced laminaribiose using the whole-cell biocatalyst LR01, which co-expressed BASP and PSLBP. A high titer of laminaribiose, up to 260 g/l, was obtained, corresponding to a substrate conversion rate of 75% (Fig. 6). To the best of our knowledge, these are the highest reported cellobiose and laminaribiose titers from sucrose and glucose. This study demonstrates a promising strategy for meeting industrial requirements for large-scale cellobiose and laminaribiose production. Furthermore, this work could assist the sugar industry to diversify the production of value-added products from sucrose and glucose by using different phosphorylases.

## Supplementary Material

kuac008_Supplemental_File

## Data Availability

The data sets generated and analyzed during the current study are available from the corresponding authors on reasonable request by permission of the institute and the department chairman.
