# Supplementary material for: Inorganic phosphate self-sufficient whole-cell biocatalysts containing two co-expressed phosphorylases facilitate cellobiose production
Source: J Ind Microbiol Biotechnol. 2022 Mar 15;49(3):kuac008. doi: 10.1093/jimb/kuac008 (PMC9142195; doi:10.1093/jimb/kuac008)
Supplement: kuac008_Supplemental_File [file kuac008_supplemental_file.docx]

**Supplementary material**

**Inorganic phosphate self-sufficient whole-cell biocatalysts containing two co-expressed phosphorylases facilitate cellobiose production**

Lei Wang^1, 2^, Peng Zheng^1, 3^, Meirong Hu^1^ , Yong Tao^1, 2^

^1^ Chinese Academy of Sciences Key Laboratory of Microbial Physiological and Metabolic Engineering, Institute of Microbiology, Chinese Academy of Sciences, Beijing 100101, China

^2^ College of Life Science, University of Chinese Academy of Sciences, Beijing 100049, China

^3^ State Key Laboratory of Food Science and Technology, Nanchang University, Nanchang 330047, China

* Lei Wang

wl8893@163.com

* Yong Tao

taoyong@im.ac.cn

* Correspondence: Institute of Microbiology, Chinese Academy of Sciences, No. 1 West Beichen Road, Chaoyang District, Beijing 100101, China. E-mail: wl8893@163.com; taoyong@im.ac.cn. Tel./Fax: +86-10-64807798; +86-10-18322693935.


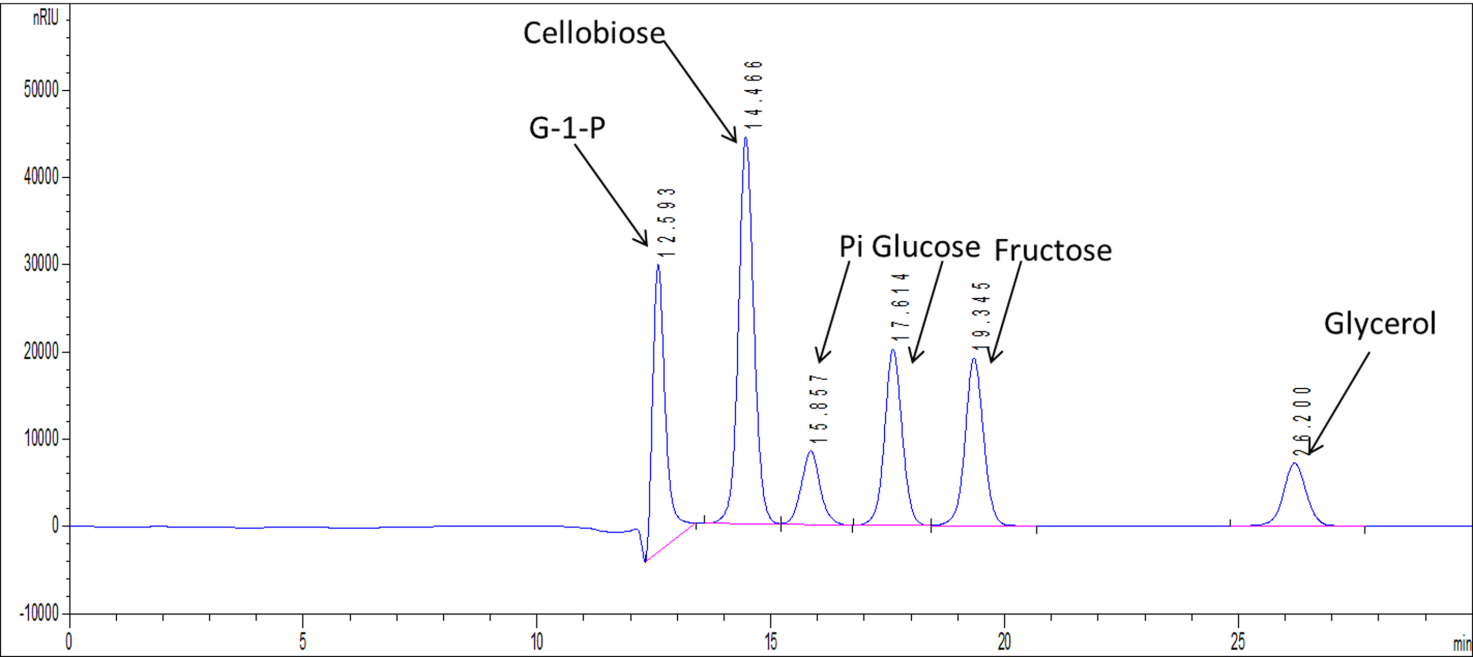


**Fig. S1** HPLC chromatograms of standard samples. The x-axis shows retention time and the y-axis shows the refractive index RID detector signal. The retention times of G-1-P, cellobiose, Pi, glucose, fructose, and glycerol were 12.593min, 14.466min, 15.857min, 17.614min, 19.345min, and 26.200min, respectively.





**Fig. S2** Time curve of cellobiose production on different substrate concentrations. Reaction conditions: OD_600 nm_=20, sucrose 0.5 M–1.0 M, glucose 0.5 M–1.0 M, sodium phosphate buffer 50 mM (pH 6.5, Pi 50 mM), 50 °C, reaction time 15 h. Error bars indicate standard deviations of three independent assays.

**
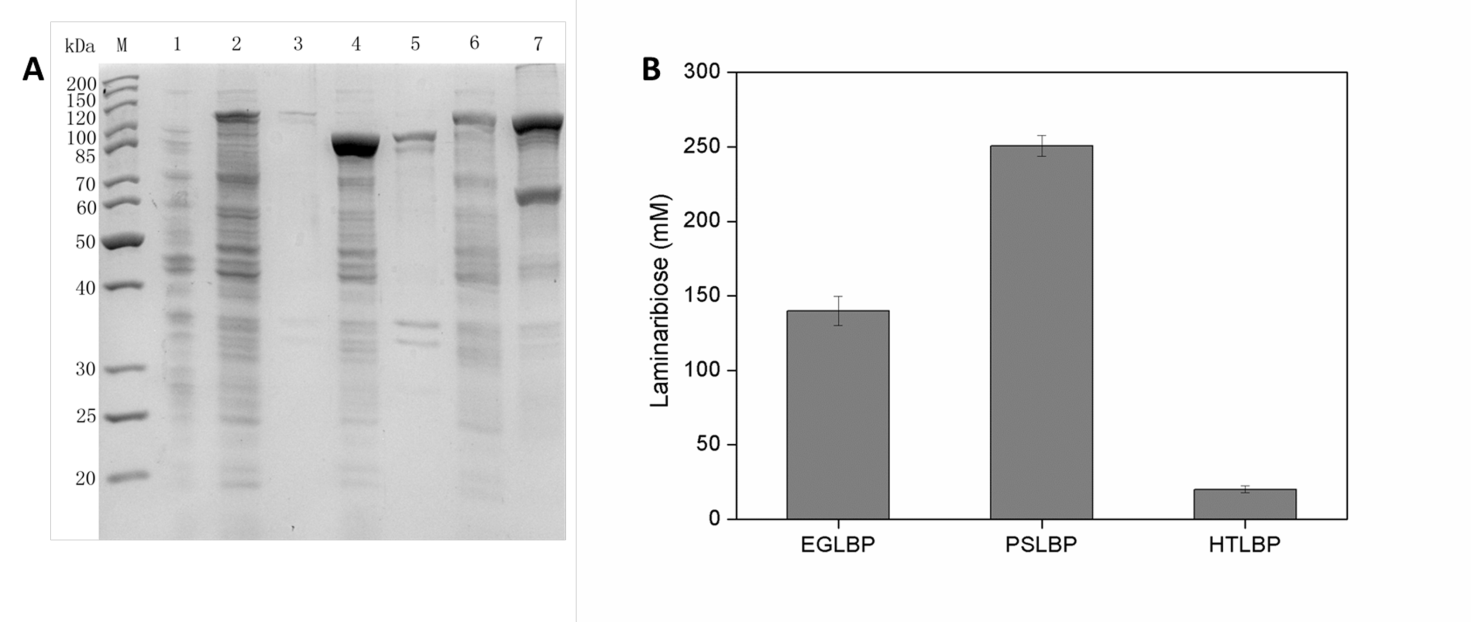
**

**Fig. S3** Expression and selection of LBP enzymes. **A** SDS-PAGE analysis of the LBP enzymes. Lane M, protein marker; Lane 1, Control; Lane 2, Soluble extract of *E. coli* expressing EGLBP; Lane 3, Insoluble extract of *E. coli* expressing EGLBP; Lane 4, Soluble extract of *E. coli* expressing PSLBP; Lane 5, Insoluble extract of *E. coli* expressing PSLBP; Lane 6, Soluble extract of *E. coli* expressing HTLBP; Lane 7, Insoluble extract of *E. coli* expressing HTLBP. **B** Laminaribiose production from G-1-P and glucose by LBP enzymes from different species. Reaction conditions: biomass OD_600 nm_ = 20, G-1-P 0.5 M, glucose 0.5 M, Pi 0 mM, sodium acetate/acetate buffer (pH 5.0), 37 °C, reaction time 12 h. Error bars indicate standard deviations of three independent assays.


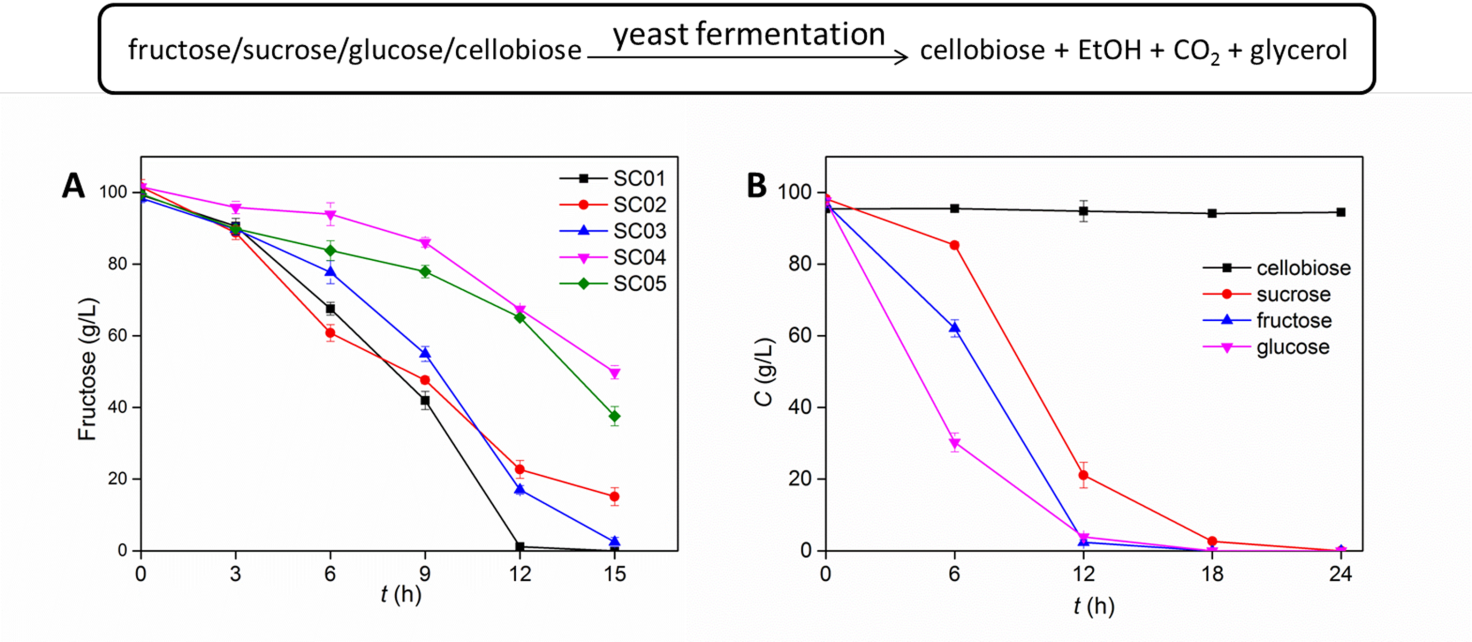


**Fig. S4** Yeast fermentation to remove fructose, sucrose, and glucose. **A** Selection of yeast strains for consuming by-product (fructose). The yeast strains SC01-SC05 were kept in our laboratory. **B** The yeast strain SC01 fermentation for cellobiose, sucrose, fructose, and glucose. Reaction conditions: biomass OD_600 nm_ = 30, sugars (cellobiose/sucrose/fructose/glucose) 100 g/L, sodium acetate/acetate buffer (pH 5.0), 30 °C, reaction time 15-24 h. Error bars indicate standard deviations of three independent assays.


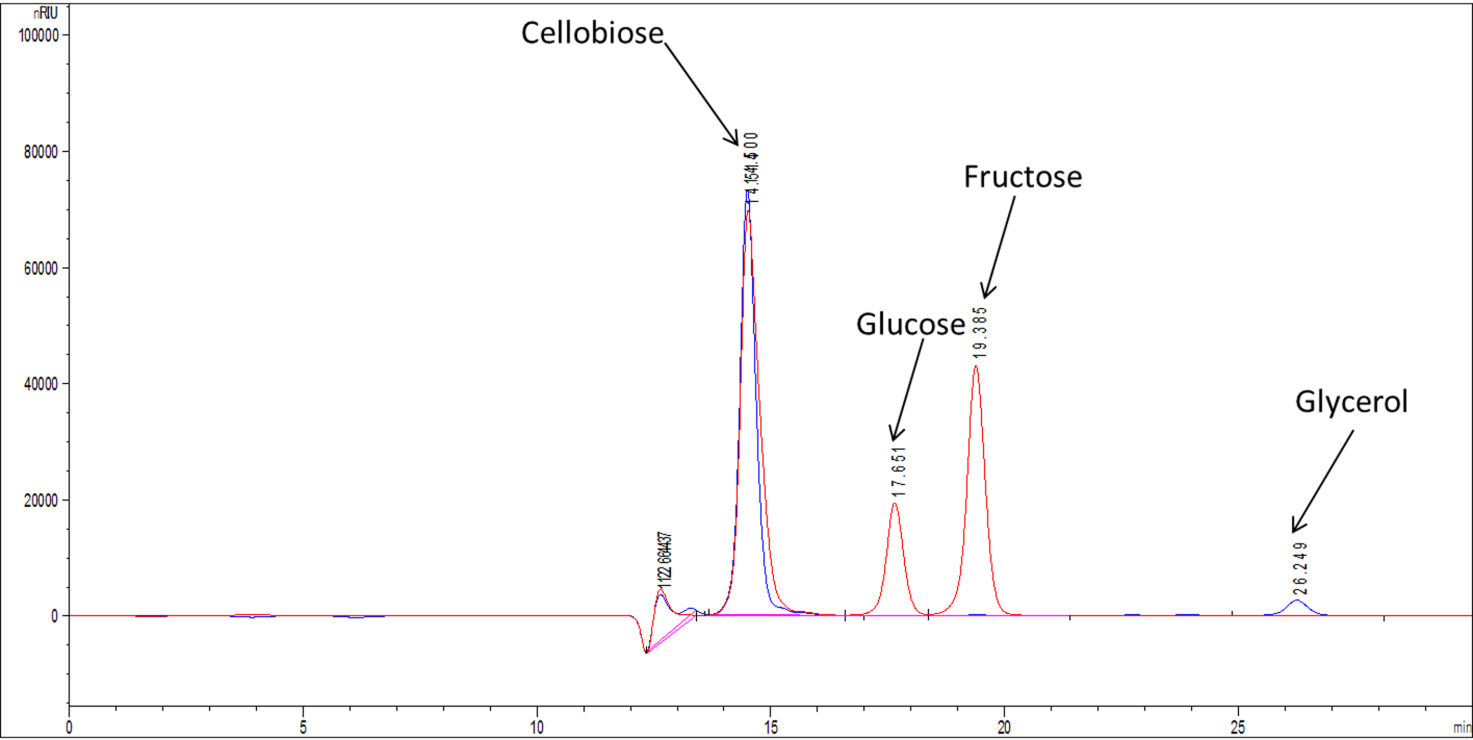


**Fig. S5** HPLC chromatograms in the production of cellobiose. The x-axis shows retention time and the y-axis shows the refractive index RID detector signal. The red RID signal shows the reaction result by CB01 treatment, and the blue RID signal shows the reaction result by yeast treatment.

**Table S1** Primers used in this study.

| Primers names | Sequence 5′→3′ |
| --- | --- |
| CB01 F | cgtgtggaagtgaccgtataaactagtatgaaaaacaaggtgcagc |
| CB01 R | gagctcaccgaattcaccactagttcaggcgacgacaggcggattg |
| CB02 F | ccgcctgtcgtcgcctgaactagtatgcgctatggtcacttcgac |
| CB02 R | gaccgagctcaccgaattcaccactagtttatacggtcacttccac |
| LR01 F | gtcgtaatattagctaaactagtatgaaaaacaaggtgcagctc |
| LR01 R | gctcaccgaattcaccactagttcaggcgacgacaggcggattg |

**Table S2** The predicted molecular weights of SP and CBP enzymes.

| SP and CBP enzymes | Predicted molecular weight* |
| --- | --- |
| LCSP | 50.2 kDa |
| BASP | 60.0 kDa |
| BLSP | 55.4 kDa |
| CGCBP | 85.0 kDa |
| CTCBP | 86.2 kDa |

* The predicted molecular weights was determined using the ProtParam tool: <https://web.expasy.org/protparam/>

**Table S3** The purities of cellobiose by different treatment with yeast, charcoal, and vacuum evaporation.

| Treatment | Putity (HPLC)* |
| --- | --- |
| Yeast | 27.6% |
| Activated charcoal | 21.4% |
| vacuum evaporation | 70.3% |
| crystals | 95.0% |

* The purity of product was determined by HPLC in comparison with standard powder (100%).
